# Central control of autonomic functions in health and disease

**DOI:** 10.3389/fnins.2014.00440

**Published:** 2015-01-09

**Authors:** Stuart J. McDougall, Heike Münzberg, Andrei V. Derbenev, Andrea Zsombok

**Affiliations:** ^1^Florey Institute of Neuroscience and Mental Health, University of MelbourneParkville, VIC, Australia; ^2^Pennington Biomedical Research Center, Louisiana State UniversityBaton Rouge, LA, USA; ^3^Department of Physiology, Tulane UniversityNew Orleans, LA, USA

**Keywords:** thermogenesis, glucose, TRPV cation channels, hypoglycemia, vagal afferents

There is a lack of knowledge about the neurophysiology of disease states such as obesity and related disorders. Recognizing this, basic scientists are actively investigating and learning more about how the brain controls energy homeostasis from the perspective of autonomic function.

The central nervous system controls many fundamental systems including whole body metabolism, body temperature and blood pressure. Autonomic reflexes are mediated by neural pathways in the brainstem and spinal cord and generally regulate organ and system performance very rapidly (ms). Autonomic control is also mediated by specific brain regions, such as the hypothalamus, which is responsible for mid-term (min) and long-term (hours/days) regulation of internal organ systems. Importantly, autonomic reflexes are dynamic, where adaptations can alter rapid homeostatic control over longer time scales. In this respect, an understanding of the basic neurophysiology is required to subsequently discover how these processes contribute to, or are impacted by, disease states - ranging from diabetes mellitus to hypertension.

The field of autonomic neuroscience research concentrates on those neural pathways and processes that ultimately modulate parasympathetic and sympathetic output to alter peripheral organ function. In the following eBook, laboratories from across the field have contributed reviews and original research to summarize current views on the role of the brain in tuning peripheral organ performance to regulate body temperature, glucose homeostasis and blood pressure. These mechanisms include experimental approaches ranging from the whole system to synaptic levels.

One of the most basic requirements for mammalian life is the maintenance of core body temperature and as such, this factor is tightly regulated. Tupone et al. (2014) review the central nervous system nuclei and circuitry involved in brown adipose tissue thermogenesis, a sympathetically driven mechanism to increase body temperature that has been demonstrated in species from rodents to adult humans. The Morrison laboratory has been consistently at the forefront in unraveling the brain regions involved in this mechanism and in this review, the authors also discuss the potential advantages of activation or inhibition of brown adipose tissue thermogenesis for the treatment of obesity or cardiac ischemia.

A second basic requirement for life is energy availability, with glucose as the basic substrate in mammals. Verberne et al. (2014) review the current understanding of the neural pathways that control glucose homeostasis with specific emphasis on the counter-regulatory response to hypoglycemia. This mechanism highlights the coordination between endocrine and neural outflows in regulating the supply of glucose. Diepenbroek et al. (2013) present original research indicating that deep brain stimulation of the nucleus accumbens shell in rats alters blood glucose and glucagon, a mechanism that may be mediated via the lateral hypothalamus, a site that receives strong innervation from the nucleus accumbens. Such an interaction complements the central scheme presented in the review by Verberne et al. (2014). Meanwhile, Browning (2013) presents a perspective article on the role of glucose in modulating gastrointestinal vagal afferent reflex function. Figure 2 neatly and concisely summarizes the known mechanisms by which glucose impacts the viscerosensory arm of autonomic reflexes.

The site where viscerosensory information enters the brainstem is the nucleus of the solitary tract (NTS) and this region plays a crucial role in many autonomic functions. In this context, McDougal et al. (2013) highlights an important role of astrocytes in glucose homeostasis. Specifically, the authors demonstrate that cytoplasmic calcium increases in astrocytes under low glucose conditions, an effect that could not be prevented by the neurotoxin tetrodotoxin. These data suggest that astrocytes are able to directly sense changes in central glucose levels.

The dorsal motor nucleus of the vagus (DMV) is positioned downstream to vagal afferents and receives viscerosensory information via the NTS. In the original work of Jiang and Zsombok (2014), the role of a Sirtuin in the DMV in regulating energy homeostasis is investigated. The authors show that SIRT1 modulates excitatory inputs to DMV motor neurons, which relies on potassium channel modulation to increase glutamate release from presynaptic terminals.

Taken together, these studies illustrate the overlapping and integrated mechanisms that are involved in glucose homeostasis.

Two papers investigate the role of the transient receptor potential cation channel subfamily V member 1 (TRPV1) in the dorsomedial complex and both utilized temperature as a tool in their respective assay systems. First Fenwick et al. (2014) test the hypothesis that other TRP channels apart from TRPV1 contribute to the excitatory primary afferent drive to NTS neurons. In knock out TRPV1 mice, approximately 50% of NTS neurons received primary afferent input where glutamate release could be modulated by temperature, suggesting involvement of other TRP channels in this neurotransmitter release process. The authors subsequently demonstrate nodose neurons express TRPV3 and propose this channel may be involved. While one to two steps later in the reflex circuitry Anwar and Derbenev (2013) explore the role of the TRPV1 in the DMV and observe both glutamatergic and GABAergic release is modulated by TRPV1 activation. Both papers further illustrate just how heterogeneous the dorsomedial complex is and continues to challenge efforts to investigate the neurophysiology in this region.

Finally on the sympathetic output side, at the level of the rostroventrolateral medulla (RVLM), Barnes and McDougal (2014) investigate the impact of leptin in modulating arterial pressure and renal nerve activity. The authors first utilized transneuronal tracing techniques to demonstrate leptin receptor expression in tyrosine hydroxylase positive RVLM neurons that ultimately innervate the kidney cortex. Then demonstrated that leptin microinjected into the RVLM evokes a sympathoexcitatory response to increases blood pressure and renal sympathetic nerve activity. These findings indicate a possible mechanism by which hypertension develops with obesity.

From whole system to synaptic levels, this collection of work represents the diverse range of central mechanisms that contribute to the regulation of autonomic function in relation to body temperature, energy and blood pressure homeostasis. This basic research sets the foundation for understanding how the brain coordinates and modulates peripheral organ systems. Defining these neurophysiological mechanisms will facilitate the development of advanced therapeutic approaches in the treatment of autonomic related disease states into the future.

## Conflict of interest statement

The authors declare that the research was conducted in the absence of any commercial or financial relationships that could be construed as a potential conflict of interest.
